# Beyond invasive biopsies: using VASARI MRI features to predict grade and molecular parameters in gliomas

**DOI:** 10.1186/s40644-023-00638-8

**Published:** 2024-01-02

**Authors:** Nurhuda Hendra Setyawan, Lina Choridah, Hanung Adi Nugroho, Rusdy Ghazali Malueka, Ery Kus Dwianingsih

**Affiliations:** 1 Department of Radiology, Faculty of Medicine, Public Health, and Nursing, Universitas Gadjah Mada, Jl. Farmako, Kabupaten Sleman, Daerah Istimewa Yogyakarta 55281 Indonesia; 2 Department of Electrical and Information Engineering, Faculty of Engineering, Universitas Gadjah Mada, Jl. Grafika No.2, Kabupaten Sleman, Daerah Istimewa Yogyakarta 55281 Indonesia; 3 Department of Neurology, Faculty of Medicine, Public Health, and Nursing, Universitas Gadjah Mada, Jl. Farmako, Kabupaten Sleman, Daerah Istimewa Yogyakarta 55281 Indonesia; 4 Department of Anatomical Pathology, Faculty of Medicine, Public Health, and Nursing, Universitas Gadjah Mada, Jl. Farmako, Kabupaten Sleman, Daerah Istimewa Yogyakarta 55281 Indonesia

**Keywords:** Glioma, VASARI, MRI, Grade, IDH, MGMT

## Abstract

**Background:**

Gliomas present a significant economic burden and patient management challenge. The 2021 WHO classification incorporates molecular parameters, which guide treatment decisions. However, acquiring these molecular data involves invasive biopsies, prompting a need for non-invasive diagnostic methods. This study aims to assess the potential of Visually AcceSAble Rembrandt Images (VASARI) MRI features to predict glioma characteristics such as grade, IDH mutation, and MGMT methylation status.

**Methods:**

This study enrolled 107 glioma patients treated between 2017 and 2022, meeting specific criteria including the absence of prior chemotherapy/radiation therapy, and the presence of molecular and MRI data. Images were assessed using the 27 VASARI MRI features by two blinded radiologists. Pathological and molecular assessments were conducted according to WHO 2021 CNS Tumor classification. Cross-validation Least Absolute Shrinkage and Selection Operator (CV-LASSO) logistic regression was applied for statistical analysis to identify significant VASARI features in determining glioma grade, IDH mutation, and MGMT methylation status.

**Results:**

The study demonstrated substantial observer agreement in VASARI feature evaluation (inter- and intra-observer κ = 0.714 - 0.831 and 0.910, respectively). Patient imaging characteristics varied significantly with glioma grade, IDH mutation, and MGMT methylation. A predictive model was established using VASARI features for glioma grade prediction, exhibiting an AUC of 0.995 (95% CI = 0.986 – 0.998), 100% sensitivity, and 92.86% specificity. IDH mutation status was predicted with AUC 0.930 (95% CI = 0.882 - 0.977), and improved slightly to 0.933 with 'age-at-diagnosis' added. A model predicting MGMT methylation had a satisfactory performance (AUC 0.757, 95% CI = 0.645 - 0.868), improving to 0.791 when 'age-at-diagnosis' was added.

**Conclusions:**

The T1/FLAIR ratio, enhancement quality, hemorrhage, and proportion enhancing predict glioma grade with excellent accuracy. The proportion enhancing, thickness of enhancing margin, and T1/FLAIR ratio are significant predictors for IDH mutation status. Lastly, MGMT methylation is related to the longest diameter of the lesion, edema crossing the midline, and the proportion of the non-enhancing lesion. VASARI MRI features offer non-invasive and accurate predictive models for glioma grade, IDH mutation, and MGMT methylation status, enhancing glioma patient management.

## Introduction

Gliomas are the most common primary intracranial tumors, accounting for a significant proportion of malignant brain tumors. Glioblastoma is the most common histology of glioma, representing approximately 45% of all gliomas [1]. The incidence and survival rates of glioma vary across populations, and recent studies have provided updated incidence and survival data [2]. The prognosis for glioma patients remains poor, with a 5-year relative survival rate of approximately 5% for glioblastoma [1]. The economic burden of glioma is substantial, encompassing direct medical costs, non-medical costs, and indirect costs related to productivity loss. The costs associated with glioma treatment, including surgery, radiotherapy, and chemotherapy, can be substantial. Glioma survivorship also poses socioeconomic challenges, impacting patients, their families, and society as a whole [3–5].

The importance of molecular diagnosis in brain glioma according to the WHO CNS classification 2021 is significant. The 2021 WHO classification incorporates molecular parameters in addition to histology to define many tumor entities, providing a more comprehensive and precise diagnosis in the molecular era. The integration of molecular information in CNS tumor classification allows for improved diagnostic precision, better prognostic information, and the development of targeted therapies [6].

IDH mutations, particularly in IDH1 and IDH2 genes, are mutually exclusive and have significant implications for tumor behavior and patient prognosis [7]. Gliomas with IDH mutations are associated with better overall survival and response to treatment compared to IDH wild-type gliomas [8]. Therefore, the assessment of IDH mutation status is crucial for accurate glioma classification and treatment planning. MGMT methylation status is another important molecular marker in glioma diagnosis. MGMT is a DNA repair enzyme that can counteract the effects of alkylating agents used in chemotherapy. Methylation of the MGMT promoter leads to reduced MGMT expression and increased sensitivity to alkylating agents. Therefore, the determination of MGMT methylation status can guide treatment decisions, particularly in the selection of patients who may benefit from alkylating chemotherapy [9]. The Ki-67 proliferation index is a measure of cell proliferation and is commonly used as a prognostic marker in gliomas. High Ki-67 labeling index is associated with increased tumor aggressiveness and poorer prognosis [10]. The assessment of Ki-67 proliferation index provides valuable information for tumor grading and helps in determining the appropriate treatment approach.

However, to obtain these molecular marker data, patients must undergo a brain biopsy procedure. Brain biopsies are invasive procedures that carry inherent risks, such as bleeding, infection, and damage to surrounding brain tissue. The invasiveness of the procedure can lead to patient rejection or reluctance to undergo the biopsy, especially in cases where the tumor is located in critical or difficult-to-reach areas of the brain [11]. Tumors located in eloquent or deep brain regions, such as the brainstem or basal ganglia, may be difficult to access safely. In such cases, the risk of complications and damage to vital brain structures may outweigh the potential benefits of obtaining a tissue sample. Additionally, multifocal gliomas, which involve multiple areas of the brain, may require multiple biopsies to accurately characterize the tumor [11, 12].

To overcome these challenges, alternative non-invasive methods for diagnosing and monitoring gliomas have been explored. Liquid biopsies, which involve the analysis of circulating tumor DNA or other biomarkers in body fluids such as blood or cerebrospinal fluid, offer a less invasive approach for obtaining molecular information about the tumor [13, 14]. Non-invasive imaging techniques, such as magnetic resonance imaging (MRI) and spectroscopy, can also provide valuable information about tumor characteristics and grade [15, 16].

The VASARI (Visually AcceSAble Rembrandt Images) MRI features are a set of standardized descriptors used to characterize brain tumors on contrast-enhanced MRI scans. These features provide qualitative and quantitative information about the visual appearance and characteristics of the tumor, aiding in the diagnosis, grading, and prognostication of gliomas [17]. The VASARI features encompass various aspects of the tumor, including its location, shape, enhancement quality, necrosis proportion, edema proportion, and other geometric properties [18]. The standardized nature of the VASARI features allows for reproducibility and consistency in the interpretation of MRI scans, enabling interobserver agreement and facilitating multicenter collaborations [19, 20]. The use of VASARI features in structured reporting systems improves communication between radiologists, oncologists, and other healthcare professionals involved in the management of glioma patients [20]. They have been employed in machine learning algorithms and radiomics analyses to improve the accuracy of glioma grading, prognosis prediction, and treatment response assessment [21, 22]. By combining VASARI features with other imaging features and clinical data, predictive models can be developed to guide treatment decisions and patient management [23, 24].

The novel nature of molecular markers in glioma, and the potential for non-invasive imaging to predict these data, underscore the need for further exploration and validation of non-invasive diagnostic and prognostic tools. These could mitigate the limitations and risks associated with brain biopsies. Our study aims to evaluate the potential of VASARI MRI features in providing accurate and valuable information about glioma characteristics, namely glioma grade, IDH mutation status, and MGMT methylation status. We plan to develop predictive models that integrate these MRI features with other clinical data, with the goal of guiding treatment decisions and improving patient management in glioma.

## Methods

### Ethical approval

In this observational analytical study, a retrospective analysis was conducted on both magnetic resonance (MR) imaging and molecular data belonging to 107 glioma patients who underwent treatment within our institution. All datasets were effectively anonymized and subsequently incorporated into this study, aligning with a pre-established retrospective protocol approved by the Institutional Review Board (IRB) of Universitas Gadjah Mada. The IRB provided clearance under the approval number KE/FK/1182/EC/2022, and written consents were obtained from the patients involved in the study.

### Subject selection

**Fig. 1:**
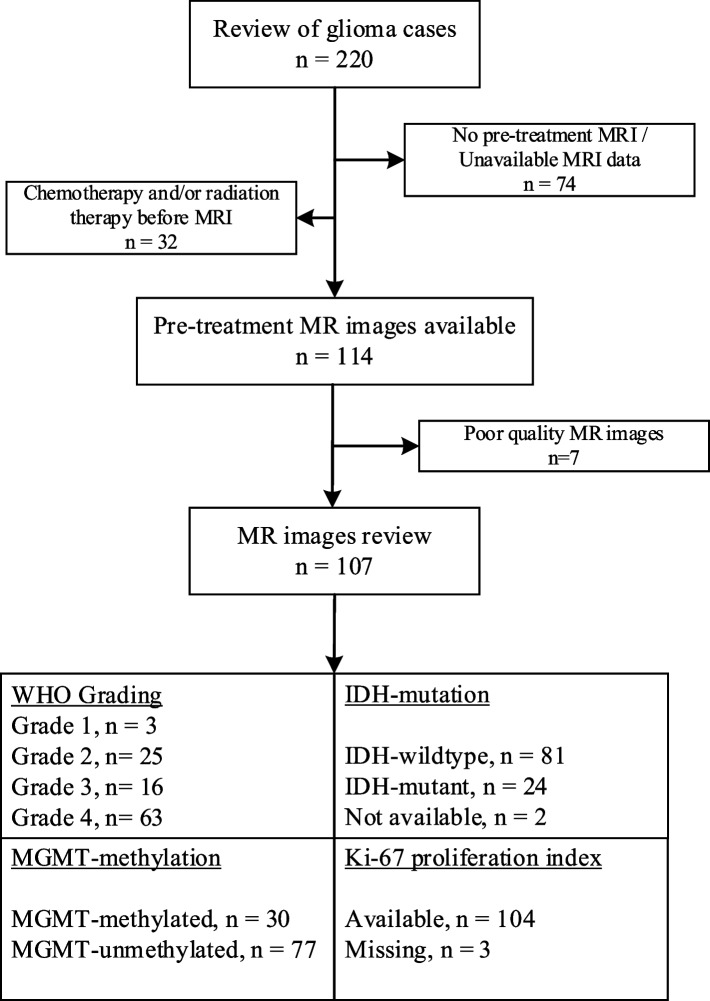
Subject selection process

Between November 2017 and November 2022, our institution treated 220 glioma patients. The inclusion criteria for this study were as follows: patients with a definitive pathologic diagnosis of glioma, presence of molecular data (IDH mutation status, MGMT methylation status, and Ki-67 proliferation index), absence of prior chemotherapy and/or radiation therapy, and availability of MR data without any severe artifacts. Medical records of 107 patients meeting these criteria were extracted from our institutional database, while imaging data were accessed from our institution's picture archiving and communication system. The detailed process of patient recruitment and the exclusion criteria are provided in Fig. 1.

### Imaging parameter and VASARI assessment

All patients underwent MR imaging using a 1.5 T Philips Multiva (Philips HealthCare, Best, Netherlands) or a 3 T Siemens Skyra (Siemens, Erlangen, Germany). The brain contrast-enhanced MRI protocol was conducted, which included axial T1-weighted images, axial T2-weighted images, axial FLAIR (Fluid-Attenuated Inversion Recovery) images, axial b_0_ and b_1000_ DWI (Diffusion-Weighted Images), and subsequent ADC (Apparent Diffusion Coefficient) images. Additionally, contrast-enhanced 3D volumetric spin echo T1-weighted images were taken after the injection of an intravenous Gadolinium-based contrast agent, Gadovist (Bayer AG, Germany), at a dose of 0.1 mmol per kilogram of body weight. Detailed parameters of the MR sequences are presented in Table 1. All imaging data were anonymized and can only be identified by the subject ID number. There is no information in the DICOM metadata that can be related to the real patient identity.

**Table 1:** MR imaging parameters used in two MR systems

**MR sequences**		**Philips Multiva 1.5 T**	**Siemens Skyra 3 T**
Axial T2-FLAIR		*Spin echo; inversion recovery*	*Spin echo; inversion recovery*
	*Slice thickness*	5 mm	4.5 mm
	*Pixel/voxel size*	0.89 × 0.89 mm	0.85 × 0.85 mm
	*Time echo*	140 ms	85 ms
	*Time repetition*	9000 ms	8000 ms
	*Inversion time*	2700 ms	2372 ms
	*Acquisition matrix*	256 × 256	256 × 256
Axial T1-WI		*Spin echo*	*Spin echo*
	*Slice thickness*	5 mm	4.5 mm
	*Pixel/voxel size*	0.71 × 0.71 mm	0.68 × 0.68 mm
	*Time echo*	15 ms	11 ms
	*Time repetition*	678 ms	1800 ms
	*Acquisition matrix*	320 × 320	320 × 320
Axial T2-WI		*Spin echo*	*Spin echo*
	*Slice thickness*	5 mm	4.5 mm
	*Pixel/voxel size*	0.34 × 0.34 mm	0.49 × 0.49 mm
	*Time echo*	120 ms	111 ms
	*Time repetition*	4000 ms	5000 ms
	*Acquisition matrix*	672 × 672	448 × 392
Axial 3D T1-WI post-contrast administration		*Spin echo volumetric*	*Spin echo volumetric*
	*Slice thickness*	1.2 mm	0.9 mm
	*Pixel/voxel size*	0.7 × 0.7 mm	0.89 × 0.89 mm
	*Time echo*	9.3 ms	11 ms
	*Time repetition*	400 ms	700 ms
	*Acquisition matrix*	352 × 352	256 × 256
Axial DWI & ADC		*Echo planar imaging*	*Echo planar imaging*
	*Slice thickness*	5 mm	4 mm
	*Pixel/voxel size*	0.7 × 0.7 mm	1.72 × 1.72 mm
	*Time echo*	72 ms	59 ms
	*Time repetition*	3500 ms	5870 ms
	*Acquisition matrix*	336 × 336	128 × 128
	*b-value*	0 & 1000	0 & 1000

*FLAIR* Fluid-Attenuated Inversion Recovery, *T1-WI* T1-Weighted Images, *T2-WI* T2-Weighted Images, *DWI* Diffusion-Weighted Images, *ADC* Apparent Diffusion Coefficient

The visual radiomic features were assessed by two radiologists (each with experience and exposure to brain glioma cases for 5 years) in a room with adequate lighting, using a diagnostic monitor, and the OsiriX DICOM Viewer software version 8.5 (Pixmeo, Switzerland). Both radiologists were blinded to the patient's basic information, including histopathological results and molecular data. Initially, both radiologists independently reviewed the entire dataset and assessed the visual radiomic features using VASARI parameters [18]. Differences of opinion were resolved through discussion, resulting in a final common assessment. This consensus-building step occurred after the initial interpretations but prior to the calculation of Kappa values comparing the two radiologists. However, for intra-observer agreement, only one radiologist repeated the assessment 4 weeks later.

Based on the VASARI assessment, a brain glioma has several components: necrotic areas, enhancing solid areas, non-enhancing solid areas, and edema areas. Necrotic areas are defined as areas that are hypointense on T1-WI compared to normal brain parenchyma, hyperintense on T2-WI, and do not enhance after contrast administration. Enhancing solid areas are defined as areas that are hypointense or isointense on T1-WI compared to normal brain parenchyma, hypointense or isointense on T2-WI, and enhance after contrast administration. Non-enhancing solid areas are defined as areas that are hypointense or isointense on T1-WI compared to normal brain parenchyma, hypointense or isointense on T2-WI, but do not enhance on T1-WI post-contrast administration and do not have signals corresponding to necrotic areas. The edema area is the area outside the boundary of the necrotic and solid areas, appearing hypointense on T1-WI compared to normal brain parenchyma, hyperintense on T2-WI, but does not enhance after contrast administration. The edema area also features vasogenic edema characteristics such as a finger-like appearance or pseudopods. Hemorrhage generally appears as hyperintensity on T1-WI and hypointensity on T2-WI, which can be confirmed with hypointense appearance on SWI (susceptibility-weighted imaging). Diffusion characteristic assessment is performed on DWI b1000 and ADC images, considering the presence of intratumoral hemorrhage products and the possible T2-shine through effect. In total, there are 27 MRI features evaluated, namely tumor location, side of tumor epicenter, eloquent brain areas, enhancement quality, proportion of enhancing tumor, proportion of non-enhancing tumor (nCET), proportion of tumor necrosis, cysts, multifocal or multicentric lesions, T1/FLAIR ratio, thickness of the enhancing margins, definition of the enhancing margins, definition of the non-enhancing margins, proportion of edema, edema crossing midline, hemorrhage, diffusion characteristics, pial invasion, ependymal extension, cortical involvement, deep white matter invasion, nCET crosses midline, CET crosses midline, satellites, calvarial remodeling, longest diameter of FLAIR abnormality, and perpendicular diameter of the longest FLAIR abnormality.

### Pathological and molecular assessment

All tissue samples were obtained from biopsy results or using fresh surgical tissues preserved in formalin-fixed paraffin-embedded (FFPE) blocks. All specimens were evaluated for classification according to the WHO 2021 Central Nervous System Tumor classification by a pathologist. The determination of the degree of brain glioma was based on morphological criteria. To confirm that the specimen was indeed a brain glioma, an immunohistochemical examination was performed on the glial fibrillary acidic protein. The evaluation of the Ki-67 labeling index was conducted using the mean method by two pathologists without knowledge of the tumor grade and the results of other doctors' interpretations. The IDH mutation status examination used the immunohistochemical method, the RFLP (Restriction Fragment Length Polymorphism) method, or PCR from FFPE blocks or fresh tissue, in succession if earlier results were inconclusive. The MGMT methylation status examination was performed using the immunohistochemical method and qRT-PCR. The techniques used have been validated and reported in previous studies [25–27].

### Statistical analysis

All statistical analyses were conducted using Stata software version 17 (StataCorp, College Station, TX, USA). The basic characteristics of patients in the low-grade and high-grade glioma groups, IDH mutation status, and MGMT methylation were evaluated using the Chi-Squared Test and Independent Samples T-Test, accordingly. Cross-validation Least Absolute Shrinkage and Selection Operator (CV-LASSO) logistic regression method was used to identify which of the 27 VASARI features have significant predictive value in determining the grade of glioma, IDH mutation status, and MGMT methylation status. During the process of cross-validation, data are continually split into training and validation subsets. The model is trained on the training subset, and the prediction error is computed using the validation subset. This procedure aids in determining the best value for λ, that maximizes the model's predictive accuracy, meaning it reduces the estimated mean-squared prediction error to its lowest possible value (λ_opt_) [28]. The Hosmer–Lemeshow test was performed to assess the goodness-of-fit of the model. A tenfold cross-validation test was conducted by evaluating the standard deviation of the cross-validated mean AUC. The performance of the predictive model will be visualized with the receiver operating characteristic curve (ROC) and evaluated using the area under the curve (AUC). The optimal cut-off point will be selected to obtain the best sensitivity, specificity, and accuracy of the model.

## Results

### Observer agreement

In assessing both inter-observer and intra-observer agreement, we utilized Cohen's kappa coefficient. For inter-observer agreement, the kappa coefficient ranged from 0.714 to 0.831, which indicates substantial agreement between different observers across all VASARI features. As for the intra-observer agreement, evaluations were conducted four weeks apart, yielding a kappa coefficient of 0.910. This falls within the 'almost perfect' agreement category, highlighting that individual observers were highly consistent in their evaluations across different time points.

### Subject characteristics

In our study, the subjects' characteristics varied across sex, age, and Ki-67 proliferation index. We further categorized these characteristics according to glioma grade, IDH mutation status, and MGMT methylation (Table 2). When examining the glioma grade, we noticed significant differences across various patient characteristics. Age demonstrated a pronounced association with glioma grade. High-grade gliomas were more common in older patients (50.35 ± 13.90 years old), while younger patients were more likely to be diagnosed with low-grade gliomas (33.32 ± 12.44 years old, *p* < 0.001). High-grade gliomas exhibited significantly higher means of the Ki-67 proliferation index compared to low-grade gliomas (24.86 vs 7.12, respectively, *p* < 0.001). The distribution of sex did not vary significantly between low-grade and high-grade glioma patients (*p* = 0.451).

**Table 2:** Subject characteristics relative to glioma grade, IDH mutation, and MGMT methylation

a. Subject characteristics relative to glioma grade			
	**Low Grade (*n*** **= 28)**	**High Grade (*n*= 79)**	***p*-value**
Sex (male)	14 (50%)	46 (58.22%)	0.451
Age (years)	33.32 (12.44)	50.35 (13.90)	< 0.001*
Ki-67^b^	7.12 (13.61)	24.86 (19.09)	< 0.001*
b. Subject characteristics relative to IDH mutation status^*a*^			
	**Mutant (*n*** **= 24)**	**Wildtype (*n*= 81)**	***p*-value**
Sex (male)	17 (70.83%)	42 (51.85%)	0.254
Age (years)	37.42 (10.54)	48.39 (15.99)	0.002*
Ki-67^b^	16.61 (18.01)	21.75 (19.86)	0.261
c. Subject characteristics relative to MGMT methylation status			
	**Methylated (*n*= 30)**	**Unmethylated (*n*= 77)**	***p*-value**
Sex (male)	16 (53.33%)	44 (57.14%)	0.721
Age (years)	48.30 (11.4)	44.96 (16.71)	0.317
Ki-67^b^	15.14 (18.05)	22.32 (19.67)	0.087

Chi-Squared Test and Independent Samples T-Test were utilized as appropriate. Data are presented as number (percentage) or mean (standard errors)

*IDH* Isocitrate dehydrogenase, *MGMT* Methylguanine DNA‐methyltransferase

^*^denotes statistically significant *p* value of less than 0.05

^a^data missing (*n* = 2)

^b^data missing (*n* = 3)

In terms of IDH mutation status, significant variations were observed across age. IDH-wild types were found more often in older patients than in younger ones, with a mean age difference of 48.39 vs 37.42 (*p* = 0.002). Upon comparing IDH mutation status with MGMT methylation, we found a significant difference in proportion; most of the MGMT-unmethylated gliomas were also IDH-wild type (*p* < 0.001). Sex and Ki-67 proliferation index did not differ significantly between IDH-mutant and IDH-wild type groups (*p* = 0.254 and *p* = 0.261, respectively). Similar patterns were not observed with MGMT methylation status, with no statistically significant difference observed among sex (*p* = 0.721), age (*p* = 0.317), and Ki-67 proliferation index (*p* = 0.087).

### VASARI and grade prediction

**Fig. 2:**
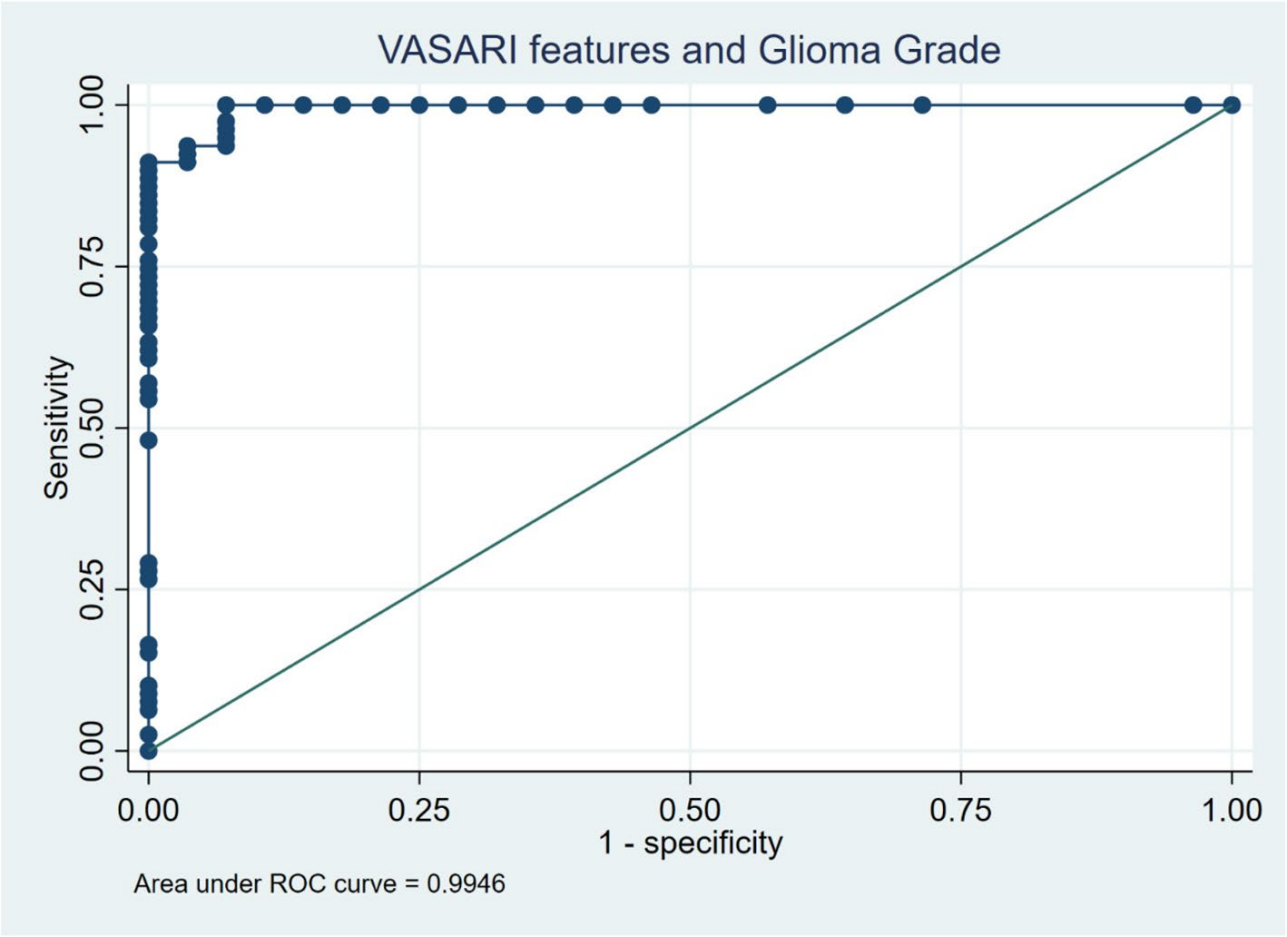
Receiver operating characteristic (ROC) curve with an area under the curve (AUC) value of 0.9946 for the model prediction of glioma grade based on VASARI features

The logistic regression procedure using the cross-validation LASSO technique on the 27 VASARI features to predict glioma grades resulted in 8 significant predictive features that have non-zero coefficients (Table 3). The features included in the glioma grade prediction model based on VASARI features are enhancement quality (f4), proportion enhancing (f5), proportion necrosis (f7), presence of cyst (f8), T1/FLAIR ratio (f10), presence of hemorrhage (f16), and diffusion characteristics (f17). This prediction model has good Hosmer–Lemeshow goodness-of-fit (*p* = 0.997) and robustness, with a standard deviation of 0.040 in the tenfold cross-validation test. The ROC curve depicts the discriminative ability of this model, with an AUC value of 0.995 (95% CI = 0.986 – 0.998) (Fig. 2). The sensitivity of this model is 100%, with a specificity of 92.86%, and an accuracy of 98.13%. Adding the predictor variable age did not significantly increase the AUC.

**Table 3:** VASARI features predicting high grade glioma yields 8 significant features with non-zero coefficients

**Selected VASARI features**	**Logistic LASSO coefficient**	**Category**	**OR (95% CI)**	***p*-value**
f4–enhancement quality	1.090	(marked/avid)	20.70 (4.782—89.607)	< 0.001
f5–proportion enhancing	1.549	(34–67%)	11.48 (1.302—75.159)	0.028
f7–proportion necrosis	0.719			
f8–cysts	-1.004			
f10–T1/FLAIR ratio	0.503	(infiltrative)	41.99 (4.603—383.215)	0.001
f13–definition of the non-enhancing margin	0.449			
f16–hemorrhage	0.650	(yes)	13.57 (4.553—40.442)	< 0.001
f17–diffusion characteristics	0.966			

Categories (within parentheses) have significant odds ratios compared to their base categories for predicting high grade glioma

*VASARI* Visually AcceSAble Rembrandt Images, *OR* odds ratio, *FLAIR* fluid-attenuated inversion recovery, *LASSO* (Least Absolute Shrinkage and Selection Operator)

### VASARI and molecular profile

**Fig. 3:**
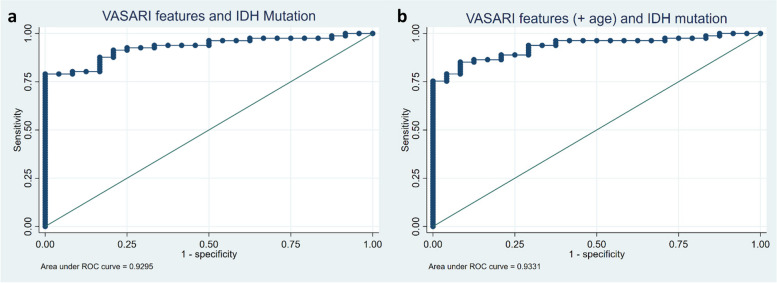
Receiver operating characteristic (ROC) curve with (**a**) an area under the curve (AUC) value of 0.930 for the model prediction of IDH mutation status based on VASARI features and (**b**) AUC value of 0.933 with the addition of age-at-diagnosis variable

We attempted to identify which VASARI features could serve as reliable predictors for IDH mutation status and MGMT methylation. IDH-mutant was established as the base category, enabling predictor features to be used for estimating the likelihood of patients with certain MRI characteristics having IDH-wildtype status. LASSO regression cross-validation techniques identified 12 predictor features with non-zero coefficients as predictors of IDH mutation status (Table 4). This IDH mutation prediction model demonstrated good Hosmer–Lemeshow goodness-of-fit (*p* = 0.771) and robustness, with a standard deviation of 0.102 in the tenfold cross-validation test. The ROC curve indicated good performance with an AUC of 0.930 (95% CI = 0.882—0.977). The addition of the predictor variable 'age-at-diagnosis' improved the AUC performance to 0.933 (Fig. 3). The best sensitivity, specificity, and accuracy of this model were 92.59%, 70.83%, and 87.62%, respectively. A deeper analysis of the categorical VASARI features revealed the specific odd ratios for each category within the VASARI features, compared to its base category (Table 4). Those features were enhancing proportion (f5) of 34–67% (OR 6.333, 95% CI = 1.854—21.638), T1/FLAIR ratio (f10) of infiltrative (OR 4.727, 95% CI = 1.587—14.074), and thickness of enhancing margin (f11) of thick/nodular (OR 6.328, 95% CI = 1.880—21.295).

**Table 4:** VASARI features predicting IDH wildtype status resulted in 12 significant features with non-zero coefficients

**Selected VASARI features**	**Logistic LASSO coefficient**	**Category**	**OR (95% CI)**	***p*-value**
f1–tumor location	0.199			
f3–eloquent brain	0.106			
f5–proportion enhancing	0.240	(34–67%)	6.333 (1.854—21.638)	0.003
f6–proportion of non-enhancing	-0.214			
f10–T1/FLAIR ratio	0.276	(infiltrative)	4.727 (1.587—14.074)	0.005
f11–thickness of enhancing margin	0.377	(thick/nodular)	6.328 (1.880—21.295)	0.003
f13–definition of the non-enhancing margin	-0.224			
f22–non-contrast enhancing crosses midline	-0.191			
f23–contrast-enhancing crosses midline	-1.077			
f24–satellites	0.037			
f29–longest diameter	-0.142			
f30–perpendicular to longest diameter	-0.143			

Categories (within parentheses) have significant odds ratios compared to their base categories for predicting IDH wildtype status

*VASARI* Visually AcceSAble Rembrandt Images, *OR* odds ratio, *FLAIR* fluid-attenuated inversion recovery, *LASSO* (Least Absolute Shrinkage and Selection Operator), *IDH* Isocitrate dehydrogenase

**Fig. 4:**
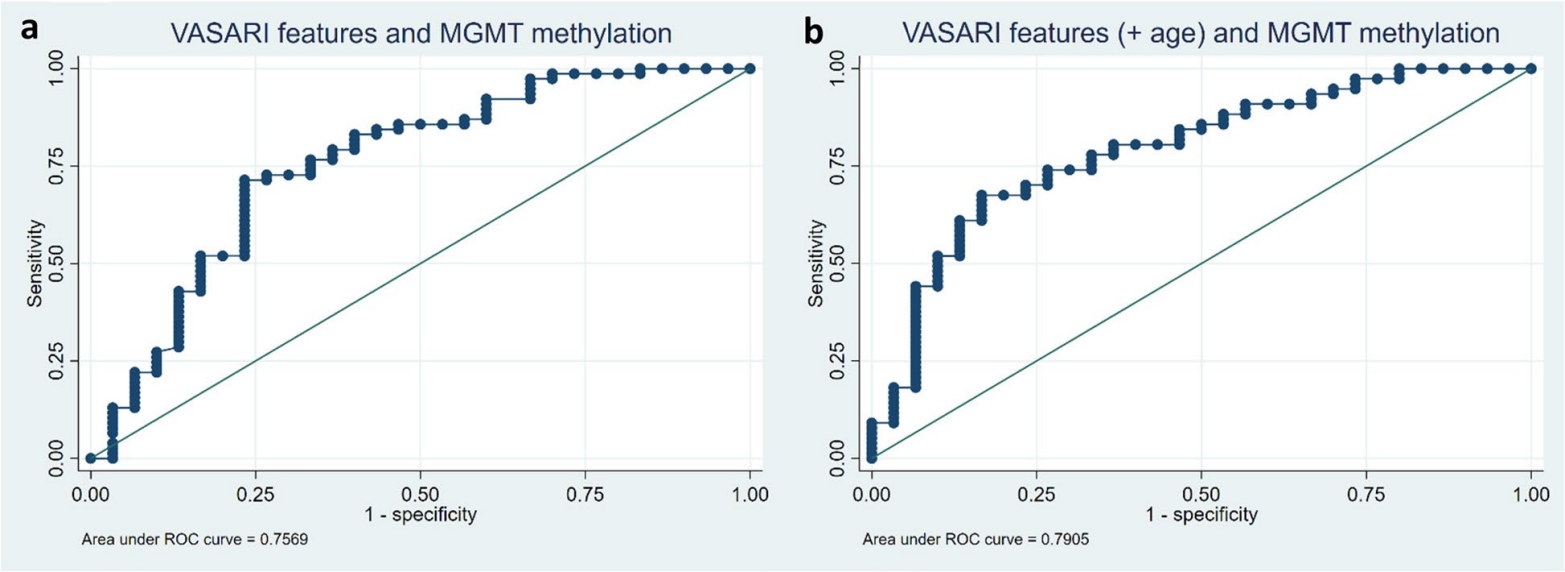
Receiver operating characteristic (ROC) curve with (**a**) an area under the curve (AUC) value of 0.757 for the model prediction of MGMT methylation based on VASARI features and (**b**) AUC value of 0.791 with the addition of age-at-diagnosis variable

MGMT methylation is another important molecular marker in glioma management. MRI through VASARI evaluation is expected to predict MGMT methylation status. MGMT-methylated was set as the base category, due to its characteristic of providing a better prognosis for patients, with predictor features being used to estimate the likelihood of a glioma being MGMT-unmethylated. Analysis using LASSO regression identified 5 predictor features with non-zero coefficients as predictors of MGMT methylation status (Table 5). This MGMT methylation prediction model demonstrated good Hosmer–Lemeshow goodness-of-fit (*p* = 0.452), with a standard deviation of 0.130 in the tenfold cross-validation test. The ROC curve indicated fairly good performance with an AUC of 0.757 (95% CI = 0.645—0.868). The addition of the 'age-at-diagnosis' variable improved the AUC to 0.791 (Fig. 4). The best sensitivity, specificity, and accuracy values were 94.81%, 33.33%, and 77.57%, respectively. Further analysis revealed which categories within the VASARI features were significant in predicting MGMT methylation status (Table 5). These categories were the proportion of non-enhancing (f6) of 68–100% (OR 0.215, 95% CI = 0.067—0.687), the presence of edema crossing the midline (f15) (OR 0.305, 95% CI = 0.125—0.742), and longest diameter (f29) (OR 0.746, 95% CI = 0.599—0.929).

**Table 5:** VASARI features predicting MGMT unmethylated status resulted in 5 significant features with non-zero coefficients

**Selected VASARI features**	**Logistic LASSO coefficients**	**Category**	**OR (95% CI)**	***p*-value**
f6–proportion of non-enhancing	-0.176	(68–100%)	0.215 (0.067—0.687)	0.009
f13–definition of the non-enhancing margin	-0.019			
f15–edema crosses midline	-0.351	(yes)	0.305 (0.125—0.742)	0.009
f25–calvarial modeling	-0.399			
f29–longest diameter	-0.084		0.746 (0.599—0.929)	0.009

Categories (within parentheses) have significant odds ratios compared to their base categories for predicting MGMT unmethylated status

*VASARI* Visually AcceSAble Rembrandt Images, *OR* odds ratio, *LASSO* (Least Absolute Shrinkage and Selection Operator), *MGMT* Methylguanine-DNA-methyltransferase

## Discussion

In this study, we attempted to create a predictive model from VASARI features to predict glioma grade, IDH mutation status, and MGMT methylation. Not every glioma patient at our institution undergoes molecular examinations, resulting in 107 patients being eligible for this study. This number is too small to form separate training and testing datasets. The large number of VASARI features also resulted in a high-dimensionality data profile, which poses its own challenges for standard regression statistical analysis. We implemented the cross-validation Least Absolute Shrinkage and Selection Operator (CV-LASSO) logistic regression technique to overcome this issue [28].

Patient age has a significant influence on the distribution of glioma grades. In our study, the average age of low-grade gliomas was 33.32 years, while that of high-grade gliomas was 50.35 years (*p* < 0.001). These data align with various other studies, which found that the grade of brain gliomas tended to increase with the patient's age, with grade IV gliomas patients having a significantly higher mean age than those with grades I and II [1, 29]. No definite mechanisms have been established regarding the influence of age on glioma grade, but several theories such as cellular senescence, changes in the immune system with age, and the progression of low-grade gliomas to high-grade gliomas over time may explain this phenomenon [30, 31]. Age also has a significant relationship with IDH mutation status. In our study, patients with IDH-mutant gliomas were on average younger compared to those with IDH-wildtype gliomas (37.42 vs 48.39 years, *p* = 0.002). The IDH mutation is often one of the earliest genetic changes in the development of certain types of gliomas, including low-grade gliomas and secondary glioblastomas. These gliomas usually occur in younger patients and have a better prognosis compared to gliomas without the mutation. On the other hand, primary glioblastomas, which are typically seen in older patients and have a poor prognosis, usually do not have the IDH mutation [32, 33]. In this study, since low-grade gliomas are clearly more frequently found in the younger age group, it makes sense that IDH-mutant gliomas are also more frequently found at younger ages.

Ki-67 is a cellular marker for proliferation that is present during all active phases of the cell cycle (G1, S, G2, and mitosis), but is absent from resting cells (G0). Therefore, the presence and percentage of Ki-67 in a tissue sample are often used as measures of the growth fraction of cells. In the context of gliomas, the Ki-67 labeling index (the percentage of tumor cells positive for Ki-67) has been used as a prognostic indicator. As previous studies have demonstrated, a higher Ki-67 labeling index often correlates with higher grade gliomas and is associated with poorer prognosis [26, 34, 35]. Our study results confirmed this with significantly lower Ki-67 labeling index in low-grade glioma compared to high-grade glioma (7.12 vs 24.86, *p* < 0.001). The Ki-67 value was also lower in IDH-mutant group compared to IDH-wildtype group, although not statistically-significant (16.61 vs 21.75, *p* = 0.261). While many studies on IDH mutations in gliomas clearly show this association, [36–38] the statistical significance can vary depending on the specific study design, sample size, and the specific patient population.

### Glioma grading prediction

Determining the grade of glioma often represents the initial step in patient management. The gold standard for determining the grade still involves a brain biopsy, an invasive procedure often met with reluctance by patients and their families. The structured and systematic assessment of MRI using VASARI features is hoped to serve as a non-invasive predictor of glioma grade. In this study, eight features were part of the predictive model for determining glioma grade (Table 3). This prediction model demonstrates an exceptionally high AUC performance (0.995, 95% CI = 0.986 – 0.998), coupled with high sensitivity, specificity, and accuracy. This allows the model to reliably distinguish between low-grade and high-grade gliomas. Several studies have investigated the use of VASARI features in glioma grading [39–44]. For example, our study findings are consistent with those of Su et al., in which the imaging features of enhancement quality and proportion enhancing were significantly higher in high-grade gliomas compared to low-grade gliomas. This indicates a more severe breakdown of the blood–brain barrier in high grade gliomas [43]. High-quality enhancement (marked/avid) and enhancement proportion of 34–67% or more had odds ratios of 20.7 and 11.478 (*p* < 0.001 and *p* = 0.028, respectively), suggesting high-grade glioma.

High-grade gliomas are more aggressive and grow more rapidly than low-grade gliomas. This rapid growth often leads to the formation of new, aberrant blood vessels, a process known as angiogenesis. These newly formed vessels are typically more permeable or "leaky" than normal vessels. Consequently, when a contrast agent is administered during an MRI scan, it can more readily leak out of these abnormal vessels and into the surrounding tumor tissue, causing the tumor to appear brighter or "enhanced" in the resultant images. Moreover, high-grade gliomas often exhibit areas of central necrosis due to their rapid growth rate and inadequate blood supply. This necrosis can lead to a breakdown of the blood–brain barrier, enabling more of the contrast agent to leak into the tumor and further increase its enhancement [41, 42].

High-grade gliomas tend to have an infiltrative tumor border into the surrounding tissue. This presents as a larger area of pathological intensity in FLAIR compared to the pathology area visible on T1-weighted MRI sequences [41]. Our study supports this theory by showing that the infiltrative T1/FLAIR ratio has a high predictive value for glioma grade (OR 41.99, *p* = 0.001). The presence of intratumoral hemorrhage indirectly indicates the rapid angiogenesis process in high-grade glioma. The blood vessels formed do not have good morphology, thus are easily damaged, resulting in hemorrhage. In our study, the presence of hemorrhage was a strong predictive factor with an odds ratio of 13.57 (*p* < 0.001) for high-grade glioma, a result consistent with previous research findings [39, 40].

### IDH mutation status prediction

The 2021 WHO CNS classification system places a significant emphasis on the assessment of molecular status. One such marker being evaluated is the IDH mutation status. Mutations commonly occur in one of the two IDH genes—IDH1 or IDH2. IDH1 gene mutation is more frequently seen in gliomas and is typically characterized by a specific mutation (R132H) that leads to a single amino acid change in the enzyme's active site. This mutated IDH enzyme gains a neomorphic activity, converting alpha-ketoglutarate into 2-hydroxyglutarate (2-HG), an oncometabolite promoting cancer formation [33, 45]. Notably, gliomas with IDH mutations have a significantly better prognosis than those without these mutations, making IDH status a crucial factor in glioma classification and treatment decisions.

In our study, 12 VASARI features were found to predict IDH mutation status following selection using the LASSO regression technique (Table 4). Our predictive model performed well with a high AUC value. However, the inclusion of the age-at-diagnosis variable improved this AUC performance (0.930 vs. 0.933, Fig. 3) with a sensitivity, specificity, and accuracy of 92.59%, 70.83%, and 87.62%, respectively. Of these features, three demonstrated high odds ratios with *p*-values < 0.005, namely, proportion enhancing, T1/FLAIR ratio, and thickness of the enhancing margin.

A proportion enhancing of 34–67% or more has an OR of 6.33 (*p* = 0.003) for predicting IDH-wildtype status. According to the study by Olar et al., there is an association between the proportion of enhancing tumor and IDH mutation status in glioma. The study evaluated the role of IDH mutation status, tumor grade, and mitotic index in patient outcome in grade II-III diffuse gliomas. The results indicated that the effect of the mitotic index on patient outcome depends on IDH mutation status [46, 47]. A similar rationale could be put forward for the predictive feature thickness of the enhancing margin (OR 6.328, *p* = 0.003), where a thicker/nodular enhancing area indicates a higher proportion of enhancement, rapid tumor cell proliferation, and aggressive growth patterns. FLAIR sequences are more sensitive in showing areas with pathological signal intensities. In the case of gliomas, especially high-grade gliomas with IDH-wildtype, pathological FLAIR areas outside of the tumor enhancement boundary tend to represent tumor cell infiltration into the surrounding tissue rather than edema processes [41]. The T1/FLAIR ratio proved to be a good predictor (OR 4.727, *p* = 0.005) in distinguishing between gliomas with IDH-mutant and IDH-wildtype.

### MGMT methylation prediction

The O6-methylguanine-DNA methyltransferase (MGMT) gene plays a vital role in cellular defense against mutagenesis and toxicity from alkylating agents. The MGMT gene encodes a DNA repair enzyme, which repairs the O6-methylguanine DNA lesion caused by alkylating agents by transferring the methyl group onto itself. This direct repair process prevents transition mutations and safeguards the genome. In gliomas, the MGMT promoter methylation status is of significant interest. When the promoter region of MGMT is methylated, the expression of the MGMT enzyme is suppressed, which inhibits DNA repair and makes the tumor cells more susceptible to alkylating chemotherapy drugs such as temozolomide. Therefore, MGMT promoter methylation status is a critical prognostic and predictive marker in glioblastoma. Patients with MGMT-promoter-methylated glioblastoma typically have a better response to alkylating agent chemotherapy and have a longer overall survival compared to those with an unmethylated MGMT promoter [48, 49].

Our study results illustrate five VASARI features that hold predictive value regarding the methylation status of MGMT (Table 5). Our predictive model demonstrates adequate performance in distinguishing between MGMT-methylated and MGMT-unmethylated gliomas, with an AUC of 0.757. As with the IDH predictive model, the inclusion of the 'age-at-diagnosis' variable improves the model's performance, resulting in an AUC of 0.791 (Fig. 4), with a sensitivity, specificity, and accuracy of 94.81%, 33.33%, and 77.57%, respectively. We identified three features possessing significant odds ratios, with statistical significance at *p* < 0.05, including proportion of non-enhancing ‘68–100%’, edema crossing the midline, and the longest diameter.

The 'proportion of non-enhancing 68–100%' feature presents an OR of 0.215 (*p* = 0.009) for being an MGMT-unmethylated glioma, implying that the larger the non-enhancing area, the greater the likelihood of the glioma being MGMT-methylated. This corresponds with previous studies that identified ring-enhancing pattern and extensive, heterogeneous enhancements related to MGMT-unmethylated status [50–52]. These MRI findings may validate the theory that gliomas with MGMT-unmethylated possess intact DNA repair mechanisms, enabling continuous tumor proliferation and reduction in the effectiveness of chemotherapy agents [48, 49].

The presence of 'edema crossing the midline' holds an OR of 0.305 (*p* = 0.009) in predicting MGMT-unmethylated gliomas. This finding is consistent with another significant predictor feature, 'longest lesion diameter', measured on axial FLAIR sequences, which shows an OR of 0.746 (*p* = 0.009) for predicting MGMT-unmethylated status. This suggests that the larger the longest lesion diameter, the higher the likelihood of MGMT methylation. Few studies have discovered a relationship between gliomas crossing hemispheres and MGMT methylation. A study by Han et al. found no significant association between 'edema crossing the midline' and MGMT methylation status [50]. However, our study finds that gliomas with edema crossing the midline tend to be associated with MGMT methylation. To the best of our understanding, no theory fully explains this phenomenon; however, we propose that gliomas with MGMT methylation tend to grow more slowly and stably, without a large necrotic area. The common occurrence of extensive necrotic areas in high-grade gliomas with IDH-wildtype and MGMT-unmethylated often limits tumor expansion [46]. This slower growth may lead to larger tumor sizes, accompanied by more extensive and crossing the midline edema.

**Fig. 5:**
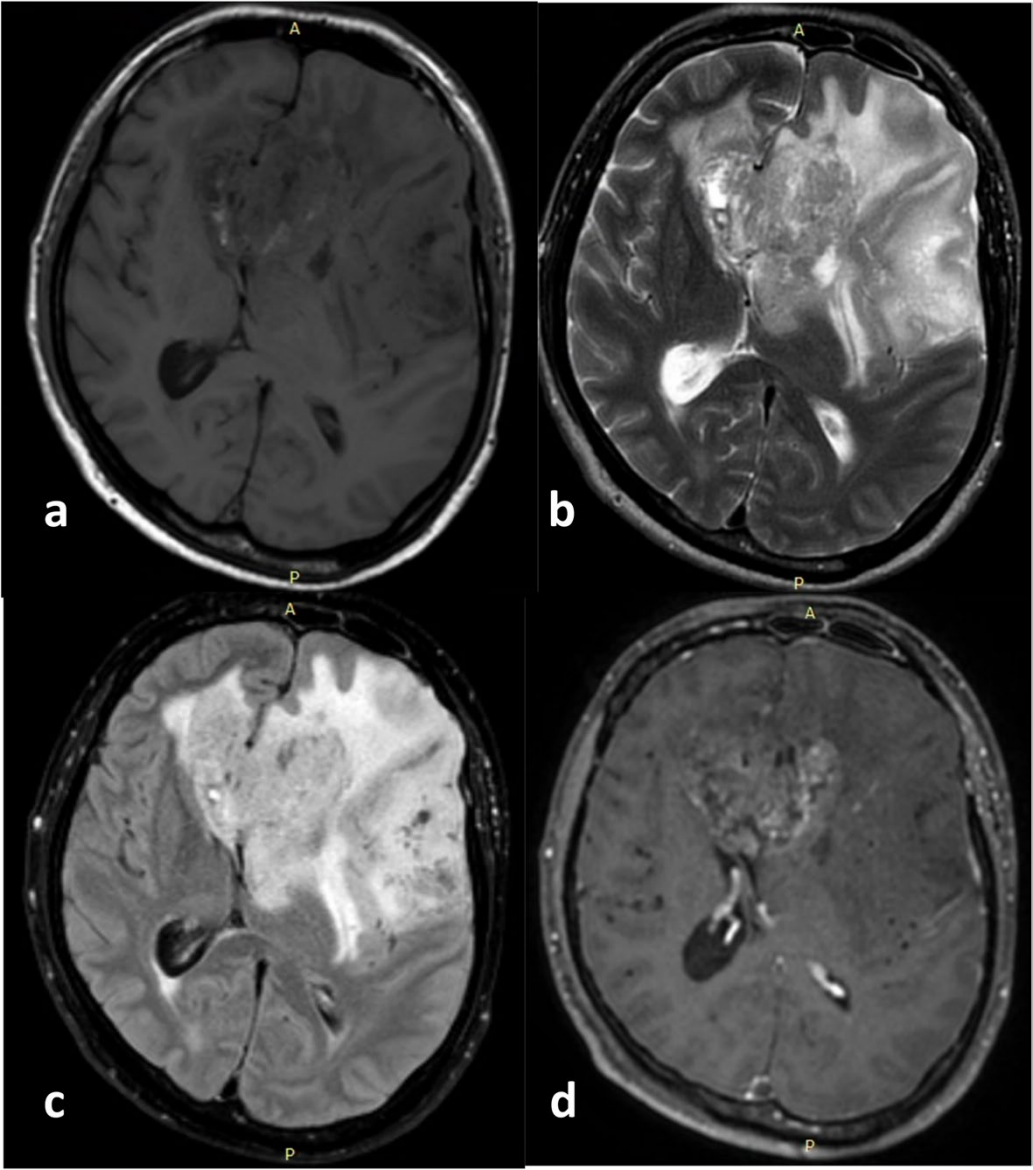
**a** Axial T1-weighted, (**b**) T2-weighted, (**c**) FLAIR, and (**d**) post-contrast administration T1-weighted MR images from a 40-year-old male with a WHO CNS Glioma Grade 4. Area of enhancement is visible that is less than 33% of the total area with pathological FLAIR signal intensity.The abnormal areas on T1 and FLAIR being approximately the same (expansive), as well as the enhancement having a vague and thin thickness. Despite the high-grade histopathological appearance, this glioma is IDH-mutant. It is predominantly solid with quite extensive non-enhancing area (68–100% of the total pathological area on FLAIR), accompanied by tumor and edema crossing the midline, and the overall large size of FLAIR abnormalities. This tumor was also proven to have methylation in its MGMT gene

**Fig. 6:**
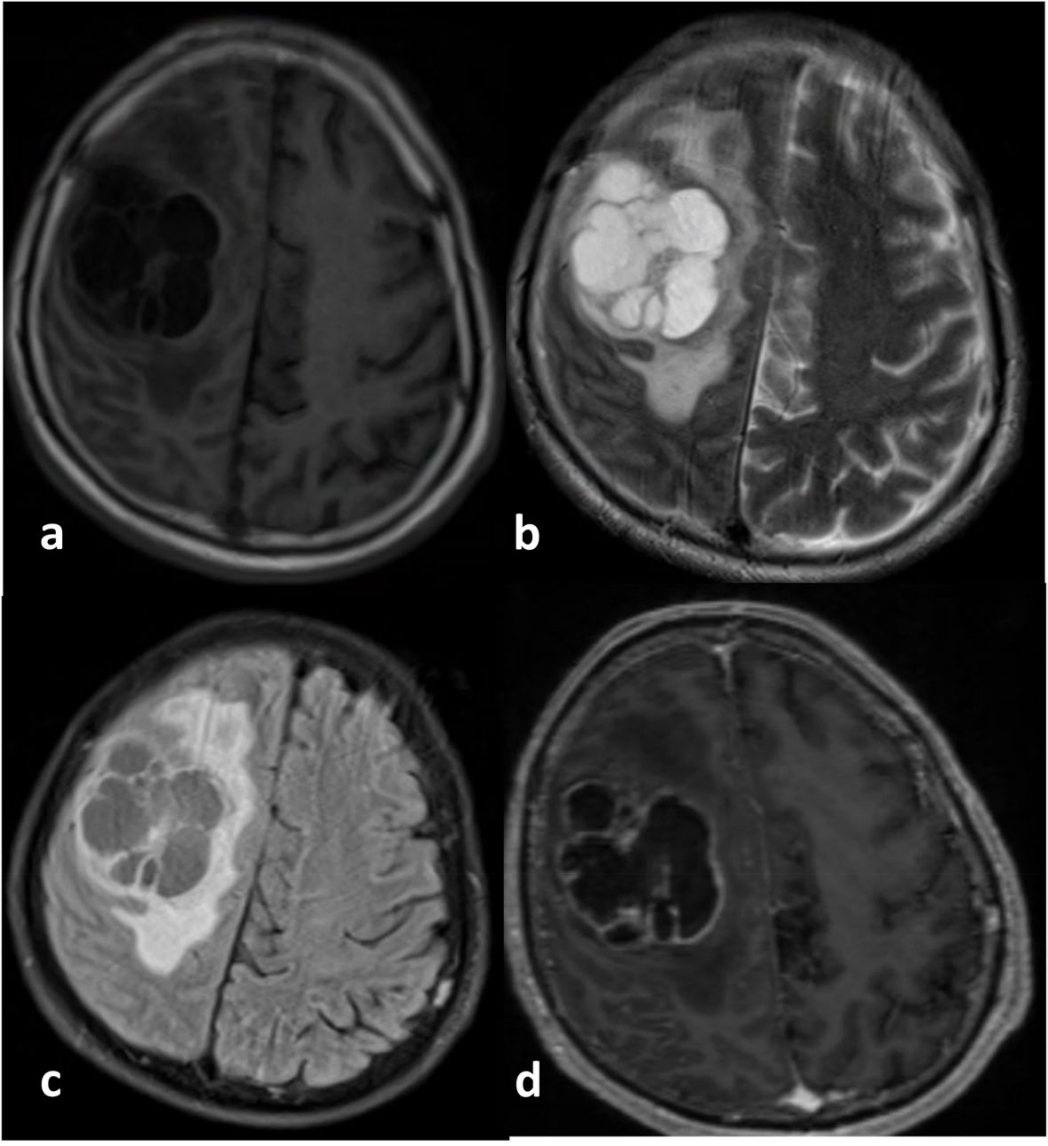
**a** Axial T1-weighted, (**b**) T2-weighted, (**c**) FLAIR, and (**d**) post-contrast administration T1-weighted MR images from a 60-year-old male with a Glioblastoma WHO CNS Grade 4, IDH-wildtype. There is a distinct and relatively thick area of enhancement on the edge of the necrotic area, with a cumulative size of about a third of the total tumor area. The abnormal FLAIR area appears slightly larger than the pathological intensity on the T1-weighted image. This glioma carries the IDH-wildtype marker. The tumor almost has no non-enhancing areas as it primarily consists of necrotic areas with a solid enhancing area at its edge. The longest diameter of the tumor size is relatively not too large, and the tumor's edema area seems limited to one hemisphere. This tumor is also MGMT-unmethylated

This study underscores the potential of non-invasive VASARI features to predict glioma grading, IDH mutation status, and MGMT methylation, leading to improved patient care. MR imaging is especially valuable in diagnosing inaccessible gliomas like intramedullary spinal cord lesions and when biopsy results conflict with clinical diagnoses, such as possible sampling errors in large intracranial masses. In areas with limited access to costly molecular marker technology, our findings present a practical alternative for glioma patient management and highlight avenues for future research on non-invasive diagnostic techniques.Two examples of brain MRIs from patients in this study, illustrating various glioma grades, IDH mutation statuses, and MGMT methylation statuses, are shown in Figs. 5 and 6.

It's worth noting that the application of VASARI features also has some challenges. For example, it typically requires the involvement of trained radiologists, and there can be variability in the interpretation of MR images. Additionally, while VASARI provides a standard language, it may not capture all relevant aspects of the imaging appearance of gliomas. The limited number of patients also makes this study unable to split all patients into two different datasets as training and testing data.

## Conclusion

Our study offers valuable insights into the potential application of MRI-based VASARI features for non-invasively predicting glioma grade, IDH mutation status, and MGMT methylation status. Our findings suggest that patient age, Ki-67 labeling index, and VASARI features are significant predictors for glioma grade, IDH mutation status, and MGMT methylation status. For high grade prediction, the most substantial predictors are the T1/FLAIR ratio, enhancement quality, hemorrhage, and proportion enhancing, with OR of 41.99, 20.7, 13.57, and 11.478, respectively. The IDH wildtype status is prominently predicted by the proportion enhancing, thickness of enhancing margin, and T1/FLAIR ratio, with OR of 6.333, 6.328, and 4.727, respectively. In contrast, MGMT unmethylated status is related to the lesion longest diameter, edema crossing the midline, and proportion of non-enhancing, with OR of 0.746, 0.305, and 0.215, respectively. Although our study faced specific limitations, such as a relatively small sample size and high-dimensionality data, it paves the way for more extensive studies to further refine and validate these predictive models, particularly using independent patient cohorts. Ultimately, this research contributes to a more profound understanding of gliomas and has the potential to enhance clinical decision-making. This could lead to personalized treatment plans and improved patient outcomes.

## Data Availability

All data were available from the corresponding author upon reasonable requests.

## Abbreviations

2-HG: 2-Hydroxyglutarate
ADC: Apparent Diffusion Coefficient
AUC: Area Under the Curve
CI: Confidence Interval
CNS: Central Nervous System
CV-LASSO: Cross-Validation Least Absolute Shrinkage and Selection Operator
DWI: Diffusion-Weighted Imaging
FFPE: Formalin-Fixed Paraffin-Embedded
FLAIR: Fluid-Attenuated Inversion Recovery
IDH: Isocitrate Dehydrogenase
IRB: Institutional Review Board
λopt: Optimal lambda
MGMT: O6-Methylguanine-DNA Methyltransferase
MRI: Magnetic Resonance Imaging
OR: Odds Ratio
PCR: Polymerase Chain Reaction
qRT-PCR: Quantitative Reverse Transcription Polymerase Chain Reaction
RFLP: Restriction Fragment Length Polymorphism
ROC: Receiver Operating Characteristic
SWI: Susceptibility-Weighted Imaging
T1-WI: T1-Weighted Imaging
T2-WI: T2-Weighted Imaging
VASARI: Visually AcceSAble Rembrandt Images
WHO: World Health Organization
